# Alpha rhythm of electroencephalography was modulated differently by three transcranial direct current stimulation protocols in patients with ischemic stroke

**DOI:** 10.3389/fnhum.2022.887849

**Published:** 2022-07-15

**Authors:** Yuanyuan Chen, Chunfang Wang, Peiqing Song, Changcheng Sun, Ying Zhang, Xin Zhao, Jingang Du

**Affiliations:** ^1^Tianjin International Joint Research Center for Neural Engineering, Academy of Medical Engineering and Translational Medicine, Tianjin University, Tianjin, China; ^2^Department of Rehabilitation Medicine, Tianjin Union Medical Centre, Rehabilitation Medical Research Center of Tianjin, Tianjin, China

**Keywords:** transcranial direct current stimulation, chronic stroke, quantitative EEG, spectral power, alpha rhythm

## Abstract

The heterogeneity of transcranial direct current stimulation (tDCS) protocols and clinical profiles may explain variable results in modulating excitability in the motor cortex after stroke. However, the cortical electrical effects induced by different tDCS protocols remain unclear. Here, we aimed to compare rhythm changes in electroencephalography (EEG) induced by three tDCS position protocols and the association between tDCS effects and clinical factors in stroke. Nineteen patients with chronic ischemic stroke underwent four experimental sessions with three tDCS protocols [anodal (atDCS), cathodal (ctDCS), and bilateral (bi-tDCS)] and a sham protocol, according to a single-blind randomized crossover design. Resting-state EEG was acquired before and after each protocol. First, a paired-sample *t*-test was used to examine the difference in spectral power between pre- and post-stimulation. Then, linear and quadratic regression models were used separately to describe the association between the clinical factors of stroke and changes in spectral power which was significantly different between pre- and post-tDCS. Finally, repeated measures analysis of variance with lesion hemisphere, stimulation protocol, and the location was performed to investigate the effects of tDCS over time. The induced effect of tDCS was mainly reflected in the alpha rhythms. The alpha power was increased by atDCS, especially low-alpha (8–10 Hz), in localized areas of the central and distant areas of the frontal and parietal lobes. Bi-tDCS also affected alpha power but in a smaller area that mainly focused on high-alpha rhythms (10–13 Hz). However, ctDCS and sham had no significant effects on any EEG rhythm. The clinical factors of time since stroke and motor impairment level were related to the change in high-alpha induced by atDCS and bi-tDCS following quadratic regression models. The above-mentioned modulation effect lasted for 20 min without attenuation. In conclusion, our findings provide evidence that the alpha rhythm of EEG is modulated differently by different tDCS protocols and that high alpha is affected by clinical characteristics such as post-stroke time and motor deficits, which is of great significance for understanding the modulation effect of different tDCS protocols on stroke and the guidance of protocols to promote motor recovery following stroke.

## Introduction

Transcranial direct current stimulation (tDCS), a non-invasive brain stimulation technique that modulates the local field potential in neural tissue and cortical excitability, has been widely used in post-stroke recovery, including motor rehabilitation. This is evidenced by its behavioral and neurophysiological effects in several previous studies (Kim et al., 2006; Wessel et al., 2015; Marceglia et al., 2016; Angulo-Sherman et al., 2017; Buch et al., 2017). However, despite its increasing application in experimental and clinical settings, results remain variable. Some studies have failed to show a positive response to stimulation in stroke patients (Hesse et al., 2011; Ochi et al., 2013; Stagg and Johansen-Berg, 2013; Powell et al., 2016; Kindred et al., 2019). Many researchers have attempted to clarify the sources of variability that affect efficiency. Factors such as the placement location of the electrodes, the polarity of the stimulation electrodes, and stimulation duration are possible reasons (Powell et al., 2016; Stephanie and Sook-Lei, 2017; Varoli et al., 2018). However, the precise mechanism by which these factors affect stimulation results remains unclear.

Among the above-mentioned sources of variability, the polarity of electrodes has proven to be especially critical in stroke patients because of the spread of functional reorganization in the post-stroke brain (Datta et al., 2011; Stephanie and Sook-Lei, 2017). Given the hypothesis that rebalancing inter-hemispheric interactions and/or restoring excitability in the ipsilesional hemisphere is beneficial for post-stroke motor recovery (Ward et al., 2003; Nudo, 2006), the present study used three protocols of tDCS position modes to modulate the excitability of the cerebral cortex in post-stroke patients: upregulating excitability of the lesional hemisphere by placing the anode over the lesioned motor cortex, downregulating the excitability of the contralesional hemisphere by placing the cathode over the unaffected motor cortex, and upregulating the excitability of the lesional cortex and downregulating the excitability of the contralesional cortex simultaneously (Nitsche and Paulus, 2000; Hummel et al., 2005, 2006; Lefaucheur et al., 2017). Some studies have found that the excitability or suppression of the brain is not a “one size fits all” approach to recovery following stroke (Nitsche and Paulus, 2000; Datta et al., 2011; Bradnam et al., 2012; Di Pino et al., 2014), which may be related to clinical characteristics of stroke patients, such as the motor impairment level or stroke period (acute, sub-acute or chronic). To date, the interactions between the modulation protocols and these factors have not been clarified.

Another key consideration when using tDCS to improve motor performance after stroke is the detection and measurement of the modulation effect of tDCS on the cerebral cortex. Recently, there has been increasing interest in exploring the local and global modulation effects of tDCS on neural plasticity using brain imaging techniques, including functional magnetic resonance imaging (fMRI) and electroencephalography (EEG). Among them, quantitative EEG (QEEG) has the advantages of low cost and high time resolution. EEG analysis in the frequency domain (spectral power analysis) provides information on rhythm oscillation of cortical electrical activity following a stroke or when induced by tDCS. Previous studies have shown that some EEG indices are sensitive to cerebral pathophysiologies following stroke and may inform clinical decision-making, including the efficacy of acute reperfusion therapies and outcome prognostication (Finnigan and Putten, 2013). Other studies have reported electrophysiological changes in EEG oscillations in healthy people during rest and task states following tDCS over the motor-related cortex (Ardolino et al., 2005; Matsumoto et al., 2010; Notturno et al., 2014).

However, most studies on EEG rhythm changes induced by tDCS have focused on healthy subjects and have obtained no consistent conclusion on the tDCS effects of different protocols. In this study, we aimed to compare the rhythm changes of EEG induced by three tDCS position protocols and the association between tDCS effects and the variability of clinical factors in stroke patients. First, we assessed the effects of the three tDCS protocols on spontaneous cortical activity through changes in the spectral power of the EEG rhythms. We then investigated the duration effects 30 min after tDCS stimulation. Finally, we attempted to determine whether the clinical characteristics (including time since stroke, location of the stroke, and level of motor impairment) impact these effects.

## Materials and methods

### Participants

Nineteen patients with chronic ischemic stroke (15 males, 4 females, mean age 56.5 ± 8.90 years, range 40–67 years, nine right-hemispheric lesions, and 10 left-hemispheric lesions) at least 3 months after subcortical cerebral infarction were included in this study. All patients were diagnosed with ischemic stroke based on MRI findings. Basic patient information is shown in Table 1. All participants were informed of all aspects of the experiment, including the possibility of minor adverse effects related to tDCS such as transient sensations of itching, burning, and prickling on the scalp. The study was approved by the Ethics Committee of Nankai University (Tianjin, China). All the participants signed a written informed consent form before commencing the experiment.

**TABLE 1:** Participant characteristics in this study.

Subject	Gender	Age(/y)	Hand	Hemisphere	Cite	Time (/m)	FM	MBI	MAL:AOU	MAL:QOM	RSS
1	Male	63	Right	Right	BG	21	55	90	38	58	1/3/2/4
2	Female	60	Right	Right	BS	9	57	80	116	116	4/3/1/2
3	Male	39	Right	Left	BS	8	59	100	57	101	4/3/2/1
4	Male	63	Right	Left	BS	13	41	70	2	4	3/4/2/1
5	Male	58	Right	Right	BG	13	20	90	1	1	2/4/3/1
6	Male	64	Right	Left	BG	20	57	100	74	114	4/1/2/3
7	Male	56	Right	Right	BG	21	50	90	7	7	4/3/1/2
8	Female	67	Right	Left	BG	21	10	65	0	0	4/1/3/2
9	Male	47	Right	Left	BG	11	57	90	74	96	3/2/4/1
10	Female	63	Left	Left	BG	6	64	100	113	115	1/2/3/4
11	Male	61	Left	Right	BG	21	61	100	150	145	2/3/4/1
12	Male	61	Right	Right	BG	24	44	100	23	23	2/3/4/1
13	Male	56	Right	Left	BG	26	60	100	90	90	4/3/1/2
14	Male	59	Right	Right	BG	5	63	100	120	121	4/2/1/3
15	Male	44	Left	Left	BG	5	46	85	39	32	2/3/4/1
16	Male	46	Right	Left	BG	5	17	80	0	0	2/4/1/3
17	Male	40	Right	Right	BG	5	63	100	112	133	4/2/3/1
18	Female	68	Right	Left	BG	4	53	85	29	37	2/4/1/3
19	Male	58	Right	Right	BG	5	7	35	0	0	1/4/3/2

*Age (year, y); Hand, Dominant hand; Hemisphere, Hemisphere affected by stroke; Cite, Cite of lesion; BG, Basal ganglia; BS, Brain stem; Time, Time following stroke(/month, m); FM, Fugl-Meyer scores; MBI, Modified Barthel Index; MAL, Motor activity log; AOU, Amount of use; QOM, Quality of movement; RSS, Randomized Stimulation Sequence of four sessions of the experiment (1 represents the anodal transcranial direct current stimulation; 2 represents the cathodal transcranial direct current stimulation; 3 represents the bilateral transcranial direct current stimulation; 4 represents the sham stimulation).*

### Experimental design

This was a single-blind, randomized, controlled crossover experiment consisting of four within-subject experimental sessions: three active conditions (anodal, atDCS; cathodal, ctDCS, and bilateral, bi-tDCS) and a sham condition. Sham stimulation served as a control to isolate the effects of current stimulation from the placebo and somatosensory effects that could arise from tDCS application. We generated a random table using the block random method through the MATLAB program to determine the implementation order of the atDCS, ctDCS, bi-tDCS, and sham conditions. The patients performed four sessions in the order shown in the randomized table and were blinded to the condition. The interval between each of the four conditions was at least 1 week.

**FIGURE 1:**
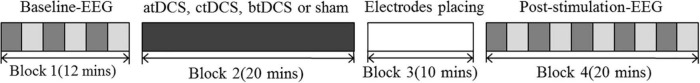
Experimental design of each session. Dark gray blocks represent the eyes-close state; Light gray blocks represent the eyes-open state. atDCS, anodal transcranial direct current stimulation; ctDCS, cathodal transcranial direct current stimulation; btDCS, bilateral transcranial direct current stimulation; Block 1: EEG signals are collected for 12 min which contains six trails with three eyes-closed states and thee eyes-opened states. The EEG signals were collected. Block 2: real (atDCS, ctDCS or btDCS) or sham tDCS is delivered for 20 min. Block 3: Electrodes of EEG acquisition were placed on the scalp, which lasts for 10 min. Block 4: EEG signals are collected for 20 min which contains 10 trials with five eyes closing states and five eyes opening states.

Each session contained four blocks: baseline EEG, tDCS, EEG electrodes, and EEG post-stimulation. All four blocks were conducted in a quiet, electrically shielded room. Patients were asked to sit and relax comfortably during the experiment. In the baseline block, patients were required to open or close their eyes according to a voice prompt produced by the E-prime software alternately every 2 min. This process lasted for 12 min (six trials with three eyes-closed and three eyes-open states). The EEG signals were collected and labeled using the E-prime software (Block 1). The patients washed off the gel and blow-dried their hair after completing the baseline recording. We then conducted one of the four tDCS protocols (atDCS, ctDCS, bi-tDCS, or sham) according to a random table for 20 min (Block 2). Electrodes for EEG acquisition were then placed over the scalp for 10 min (Block 3). Finally, patients were asked to open and close their eyes alternately (every 2 min) for 20 min (10 trials with five eyes-closed states and five eyes-open states). The EEG signals were collected concurrently (Block 4). Figure 1 shows the experimental design for each session.

### Transcranial direct current stimulation

Direct current was transferred using a saline-soaked pair of surface sponge electrodes (5 × 7 cm) and delivered by a specially developed battery-driven constant-current electrical stimulator (Neuroconn, Germany). The impedance of the electrode was kept below 1 kΩ when the DC stimulator was working. The primary motor cortex (M1) was the target area for the stimulation. C3 (left hemisphere) or C4 (right hemisphere), according to the international standard 10–20 EEG system (American Electroencephalographic Society, 1994), was defined as the position of M1. The placement of electrodes depended on the stimulation protocol and the damaged hemisphere in stroke patients. For anodal stimulation (atDCS), the anode electrode was placed over the M1 of the ipsilesional side, and the cathode electrode was placed over the lateral supraorbital as a reference. For cathodal stimulation (ctDCS), the cathode was placed over the M1 of the contralesional hemisphere and the anode was placed over the lateral supraorbital as a reference. For bilateral stimulation (bi-tDCS), the anode was placed over the M1 of the ipsilesional side and the cathode was placed over the M1 of the contralesional hemisphere. The placement of the sham stimulation was consistent with atDCS.

Patients were asked to sit quietly in a chair during stimulation. For stimulation, the current was ramped up over 8 s, held constant at 1.75 mA (current density: 0.5 A/m^2^) for 20 min, and finally ramped down over 8 s. During the sham condition, the electrodes were located at the same positions as in the anodal stimulation, but the current was supplied only for the first 46 s (8 s ramp up, 30 s of DC stimulation, and 8 s ramp down). This procedure ensured that the patients felt a tingling sensation at the beginning of stimulation (Mangia et al., 2014).

### Electroencephalography recording and processing

#### Recording

Resting-state EEG with eyes closed and eyes open was recorded in a quiet room with electromagnetic shielding. The participants were instructed to stay awake and avoid movement during the acquisition. The EEG signals were recorded using a SynAmps2 EEG system (Neuroscan Company, made by United States). An electrode cap with 62 electrodes placed in accordance with the International 10–20 position system was worn on the participant’s scalp. We used the same size electrode cap before and after the stimulation to ensure that the EEG electrodes were placed at the same place before and after tDCS. A pair of vertical electrooculogram (VEOG) electrodes and a pair of horizontal electrooculogram (HEOG) electrodes were also recorded to remove ocular artifacts in a subsequent processing step. Electrode impedance was kept below 10 kΩ. The EEG signal was amplified with a band pass of 0.1–70 Hz and sampled at 1,000 Hz. The forehead was set as the ground, and the linked earlobes were used as the reference electrodes.

#### Spectral power analysis

First, the EEG data were resampled to 250 Hz and filtered using a 0.25–45 Hz bandpass filter. Independent component analysis (ICA) was used to remove artifacts from eye movements. The EEG signals of 100 s (10–110 s) during the eyes-closed and eyes-open states of each trial (120 s) were selected for the following spectrum analysis separately. All the above processes were conducted using the EEGLAB toolbox of MATLAB software.

The EEG data from each channel and trial (100 s) were used to calculate the spectral power of the EEG rhythms. A digital fast Fourier transfer (DFFT)-based power spectrum analysis (Welch technique, Hamming windowing function) was used to compute the power spectrum density (PSD) average value of each EEG trial separately, with NFFT = 1,024, window = 256, and 50% overlap (Lias et al., 2011). Then, spectral power of delta (1–4 Hz), theta (4–8 Hz), alpha (8–13 Hz), alpha1 (8–10 Hz) and alpha2 (10–13 Hz) beta1 (13–20 Hz) and beta2 (20–30 Hz) rhythms were calculated according to the frequency bands. We calculated the average power of the three trials of Block 1 (eyes-closed and eyes-open separately) as a baseline for the pre-stimulation parameters and the average power of the five trials of Block 4 (eyes-closed and eyes-open separately) as post-stimulation parameters. For the same stimulation protocol, patients with different lesion sides (left or right) were stimulated at different locations, and 62 channels were normalized to the ipsilesional hemisphere, contralesional hemisphere, and central zone.

### Statistical analysis

To explore the effects of stimulation, a paired-sample *t*-test was applied to examine the difference in spectral power between pre- and post-stimulation. Statistical analysis was performed with eyes open and closed separately. Each frequency band and tDCS protocol were also conducted separately. Because 62 channels were performed simultaneously, we corrected the 62 comparisons using the Benjamini and Hochberg method of false discovery rates (BHFDR) to reduce the incidence of Type I errors. All data were normally distributed according to the Kolmogorov-Smirnov test.

Subsequently, regression analysis between clinical factors of stroke and the change in spectral power was performed using linear and quadratic regression models. We conducted the regression analysis as a *post hoc* analysis of the above Paired-samples *t*-test. Regression analysis was only performed in channels which is significantly different between pre- and post-tDCS. The demographics, age, sex, and lesion hemisphere were used as covariates. Clinical factors included the time after stroke, level of motor impairment reflected by activities of daily living, Modified Barthel Index (MBI), Motor Function Evaluation Scale-Fugl-Meyer scores (FM), and Motor Activity Log (MAL). The change in alpha power was described by the ratio of poststimulation to prestimulation. Regression analyses were conducted separately for each protocol.

Finally, the effects of tDCS over time were analyzed using a three-way repeated-measures analysis of variance (ANOVAs) with five post trials, two lesion hemispheres, and 62 locations (5 × 2 × 62) separately for each protocol. Before the ANOVAs, Mauchly’s test of sphericity was used to test covariance matrix sphericity. If the spherical assumption was not satisfied, the Greenhouse-Geisser method was used to adjust the degree of freedom to reduce the probability of a Type I error.

## Results

No statistically significant result (*p* > 0.05) was found in the eyes-closed state. Therefore, we focused on showing the results in the eyes-open state.

### Post-stimulation effects

**FIGURE 2:**
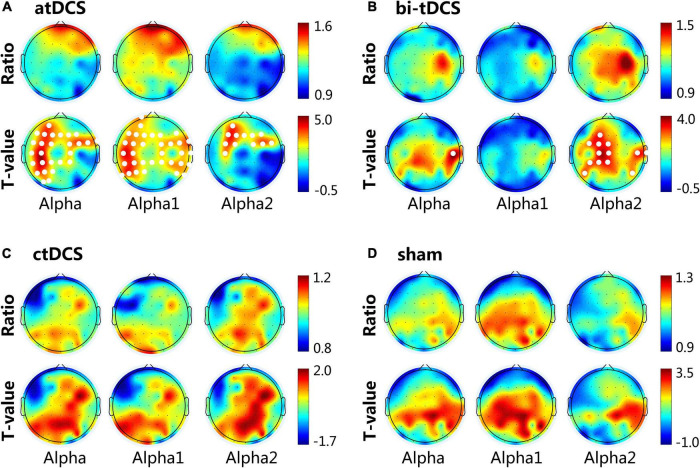
Cortical activity changing of alpha (8–13 Hz), alpha1 (8–10 Hz) and alpha2 (10–13 Hz) frequency band induced by anodal transcranial direct current stimulation (atDCS) **(A)**, bilateral (bi-tDCS) **(B)**, cathodal (ctDCS) **(C)** and sham stimulation **(D)**. For the topographical maps, the Ratio represents the spectral power ratio of post-to pre-stimulation. *T*-value represents the statistic *t*-value of the paired-samples *T*-test. The white dots represent channels that have a significant difference (*p* < 0.05) between post- and pre-stimulation. The 62 channels were normalized to the lesional hemisphere (left side), contralesional hemisphere (right side) and central.

Our results showed a significant difference between post- and pre-stimulation alpha bands in the eyes-open state. We found no significant changes in the other frequency bands after stimulation. The results of the comparison between the post- and pre-stimulation are shown in Figure 2. For the atDCS protocol, alpha power significantly increased in the prefrontal, frontal, central, and parietal lobes of the ipsilesional side and frontal and frontal-central regions of the contralesional side after stimulation. Specifically, the increase was mainly attributed to the alpha1 band; all of the channels except for the temporal of ipsilesional side, prefrontal of contralesional side, and occipital of both sides showed significant differences (*p* < 0.05) in the increase of alpha1 power. The increase in alpha2 power was only focused on the frontal and frontal-central regions of both sides (*p* < 0.05). For the bi-tDCS protocol, only the alpha2 power of post-stimulation showed an increase compared to the pre-stimulation, and a significant difference was observed in the frontal-central, central, and central-parietal regions of the lesional side and some central and temporal regions of the contralesional side (*p* < 0.05). We found no significant differences in any of the channels after ctDCS or sham stimulation.

### Association between transcranial direct current stimulation response and clinical factors

The regression analysis showed that the change in alpha2 power (ratio of post-stimulation to pre-stimulation) induced by atDCS or bi-tDCS was related to the time since stroke and Fugl-Meyer scores. The quadratic regression model was better suited for modeling the variation trend than the linear regression model for both atDCS and bi-tDCS. No correlation was found between changes in other spectral power frequencies and other tDCS protocols.

**FIGURE 3:**
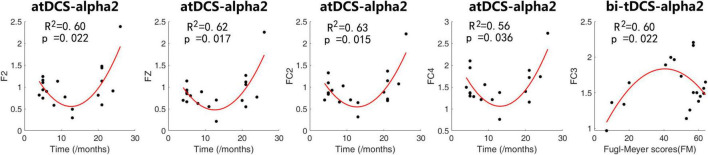
Scatter plots and fitted curves of representative channels with a clinical scale on the abscissa and alpha power’s ratio of post- to pre-stimulation. We applied quadratic fitting to these scatters with co-variables of age, sex and lesion hemisphere. The coefficient of determination *R*^2^ and *p*-value of Fisher’s *F*-test were shown in the plots.

Figure 3 shows the scatter plots and fitted curves of representative channels with a clinical scale on the abscissa and the alpha power ratio of post- to pre-stimulation on the ordinate. For atDCS, the alpha2 change ratio of contralesional frontal and frontal-central regions could be predicted by the time since stroke using a quadratic regression model. Patients with 3–6 months and longer than 20 months after stroke showed a higher alpha2 power increase than other patients, indicating a higher response to the atDCS protocol. For bi-tDCS, the alpha2 change ratio of the lesional frontal-central regions could be predicted by Fugl-Meyer scores using a quadratic regression model. The model showed that the power of alpha2 increased the most in moderately impaired patients with mild and severe impairments.

### Transcranial direct current stimulation effects of different protocols over time

**FIGURE 4:**
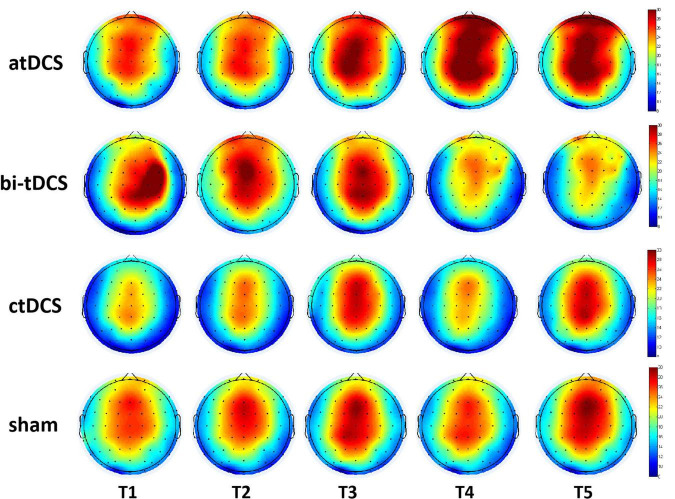
Cortical activity changing of alpha (8–13 Hz) frequency band over the 10–30 min after anodal transcranial direct current stimulation (atDCS), cathodal (ctDCS) and bilateral (bi-tDCS) and sham stimulation (sham). T1, T2, T3, T4, and T5 represents the five observation period with the eyes-open state.

For the alpha power of atDCS, repeated ANOVA results showed that there was a main effect in the within-subject factor of location [*F*(1.8, 28.8) = 8.2, *p* = 0.002*], indicating a difference in different locations. No other significant differences were found with time [*F*(2.0, 32.2) = 0.84, *p* = 0.441], lesion [*F*(1.0, 16.0) = 0.30, *p* = 0.59], time * lesion [*F*(2.0, 32.2) = 1.19, *p* = 0.32], location*lesion [*F*(1.8, 28.8) = 0.57, *p* = 0.56], time * location [*F*(4.7, 74.4) = 0.66, *p* = 0.64], and time × location*lesion [*F*(4.7, 74.4) = 0.64]. We found that the alpha power post atDCS continued to increase over this period, but it was not statistically significant (*p* > 0.05). None of the other three protocols showed any significant main or interaction effects. Figure 4 shows the change in alpha power over the observation period for the four protocols.

## Discussion

This study aimed to investigate the effects of tDCS on cortical electrical activity in patients with chronic ischemic stroke. We focused on spectral power change after three tDCS protocols (atDCS, ctDCS, and bi-tDCS) and the difference in after-effects among them. We were also interested in the after-effects of tDCS over time and the association between the change in the alpha band and the clinical characteristics of stroke patients. There were four important findings from our study: (1) The after-effect of tDCS was mainly on the alpha rhythm during the eyes-open state. (2) atDCS increased alpha power, especially the alpha1 band (8–10 Hz) in local and other areas. bi-tDCS also affected the alpha power, but in a smaller area and mainly focused on the alpha2 band (10–13 Hz). ctDCS did not affect alpha rhythm. (3) The change in alpha2 power of the contralesional frontal and frontal-central regions induced by atDCS was related to the time since stroke and of the lesional frontal-central region induced by bi-tDCS to the motor impairment level. (4) The effects of the alpha band were maintained for at least 30 min after tDCS.

The eyes-open state involves an increase in arousal and other processing capabilities compared to the eyes-closed state (Barry et al., 2007; Barry and De Blasio, 2017). According to this study, the brain is much more stimulated in the eyes-open state than in the eyes-closed state. The higher responsiveness to tDCS in the eyes-open state may be related to a higher processing capability to external tDCS stimulation than in the eyes-closed state.

The alpha band of EEG has been proven to be a brain rhythm involved in several cerebral functions, ranging from sensorimotor processing to memory formation (Klimesch, 1999; Schürmann and Başar, 2001). Ischemic stroke shows attenuation of normative, faster activity, particularly in the alpha band (8–12 Hz) (Jordan, 2004; Hirsch et al., 2013). The alpha band of stroke patients was found to be locally reduced in brain regions that are critical to the observed motor or cognitive behavioral deficits. A decrease in alpha-band synchrony was found to be related to cognitive and motor deficits in post-stroke patients (Dubovik et al., 2013). Some studies have shown that motor recovery can be predicted by increased alpha-band functional connectivity in motor-related areas (Westlake et al., 2012). Therefore, we hypothesized that an increase in the alpha band would be beneficial for stroke recovery.

Previous studies investigating changes in cortical activity after tDCS through rs-EEG power spectrum analysis mainly focused on healthy individuals and showed different responses among stimulation protocols. Some studies have shown an increase in the alpha band after atDCS in healthy individuals but not after ctDCS, which is similar to our results in stroke patients. Notturno et al. (2014) found a higher low-alpha band power post-atDCS than pre-atDCS over motor-related regions, but not for ctDCS. Spitoni et al. (2013) explored the effect of tDCS on the right posterior parietal area in healthy individuals and found that the effect was limited to the alpha band, and atDCS significantly affected the alpha band, whereas ctDCS did not elicit any modifications. This is consistent with our findings in stroke patients. However, the above effect was observed in the eyes-closed state but not in the eyes-open state, which is different from the results of this study. Differences in stimulation targets and populations may be possible reasons for this. Studies on bilateral tDCS have mainly focused on rehabilitation efficacy in stroke patients (Lindenberg et al., 2010; Bolognini et al., 2011; Lazzaro et al., 2014; Montenegro et al., 2016). A reduction in inter-hemispheric imbalance was found after a long-term effect of tDCS associated with physical therapy, according to the motor evoked potential analysis (Lazzaro et al., 2014). We found no reports of EEG power spectrum following the bi-tDCS protocol. In our study, both atDCS and bi-tDCS modulated the alpha band, but atDCS predominantly changed the low-band alpha and bi-tDCS changed the high-band alpha. Previous studies have shown that different alpha components correspond to different cognitive processes. The low-band alpha rhythm was supposed to be related to anticipatory attentional processes, and the high-alpha band would indicate task-specific visuomotor processes, according to some task-related event-related desynchronization studies (Babiloni et al., 2004). We speculate that these different changes in alpha rhythm induced by the two protocols may imply that they work in different ways.

However, the results of some studies are inconsistent with our results. In addition to the alpha band, some studies have shown power changes in other frequency bands, including the theta and beta bands, after tDCS (Ardolino et al., 2005; Pellicciari et al., 2013; Roy et al., 2014). In other studies, rs-EEG power spectrum analysis showed no difference between baseline and post-stimulation in any of the tDCS conditions (one-hemispheric tDCS or bilateral tDCS) over the dorsolateral prefrontal cortex in healthy participants (Horvath et al., 2015; Caldana et al., 2018). The confounding results may be due to the difference in stimulation target, current density, duration, and participants.

For the stimulus target area of tDCS, recent studies have shown that brain stimulation leads not only to local changes in cerebral activity in the stimulated region but also to distant changes in interconnected brain regions throughout the brain (Siebner and Ziemann, 2011; Liew et al., 2014; Roy et al., 2014), which is consistent with our results. In addition to the local target area, we found that the alpha power of some distant areas, including the frontal and parietal areas, increased after atDCS and bi-tDCS. In addition, the influence of atDCS is more widespread than that of bi-tDCS.

For the clinical factors affecting modulation results, previous studies have found that tDCS stimulation efficacy may vary with time after stroke, nature and location of the stroke, and level of motor impairment (Lindenberg et al., 2010; Chen and Schlaug, 2016; Stephanie and Sook-Lei, 2017). Our study showed that the clinical characteristics of stroke were mainly related to changes in the high-alpha band post-atDCS and bi-tDCS. Regression analyses confirmed that individuals’ response to a high-alpha power change to atDCS could be predicted from their time after stroke. Stroke patients with 3–6 months and longer than 20 months since stroke showed a higher alpha power increase than other subjects, indicating a higher response to the atDCS protocol. For bi-tDCS, high alpha power increased the most in moderately impaired patients with respect to mild and severe impairments, implying that patients with moderate motor impairment were more susceptible to this type of protocol. As plasticity processes vary with different phases or degrees of stroke, the effects of tDCS may also interact with these processes. Studies have suggested that patterns of neural recovery may differ among individuals based on the severity of their stroke (Kollen et al., 2011; Feng et al., 2016). The quadratic regression model was better suited to model the variation trend than the linear regression model for both atDCS and bi-tDCS, indicating a complicated relationship between the clinical factors and EEG parameters. Our results may help explain the variable rehabilitation efficacy of tDCS in stroke patients with different clinical profiles.

For the duration effect of tDCS, Mangia et al. (2014) reported increased alpha power during and after atDCS, which persisted for 12 min without attenuation. Spitoni et al. (2013) reported that the strongest change in alpha power occurred in the first 2 min after atDCS ended, and the effect diminished systematically and was effective for approximately 8 min. We missed the first 10 min of EEG information after stimulation because of the electrode placement. Therefore, only 10–30 min of EEG signals after stimulation were analyzed in the present study. Alpha power remained at a higher level than that of the pre-stimulation and did not change in the observed time range after stimulation, indicating that the effect was maintained for at least 30 min with no significant attenuation.

Our study has some limitations. As references to EEG changes induced by tDCS in stroke patients are limited, we discussed some of our findings based on healthy subjects, which may not be readily compared. In addition, the present study only found a change in alpha power induced by tDCS. A longitudinal analysis is needed to verify the correlation between motor improvement and changes in alpha power. Finally, although we did not find any difference in spectral power between pre- and post-stimulation in the ctDCS protocol, this does not mean that ctDCS does not affect cortical activity. We plan to attempt other methods, such as network connectivity or non-linear dynamic analysis, to explore the performance of cortical electrical activity after ctDCS and other protocols in our future research.

## Conclusion

The study provides evidence that the alpha rhythm of EEG is modulated differently by different tDCS protocols and that the high-alpha band is affected by clinical characteristics such as post-stroke time and motor deficits, which is of great significance for understanding the modulation effect of different tDCS protocols on stroke and the guidance of protocols to promote motor recovery following stroke.

## Data Availability Statement

The EEG data and clinical characteristics of stroke patients are restricted by the Tianjin Union Medical Centre to protect patients’ privacy. Data are available from the corresponding author of the article for researchers who meet the criteria for accessing confidential data.

## Ethics statement

The studies involving human participants were reviewed and approved by the ethics committee of Nankai University. The patients/participants provided their written informed consent to participate in this study. Written informed consent was obtained from the individual(s) for the publication of any potentially identifiable images or data included in this article.

## Author contributions

YC and CW: EEG data analysis, writing of the manuscript, and making tables and figures. PS: recruiting and assessing participants and collecting data. CS: statistical analysis. YZ: recruitment and organization. XZ: conception, supervision, and programming. JD: supervision. All authors have read and approved the final manuscript.

## Conflict of Interest

The authors declare that the research was conducted in the absence of any commercial or financial relationships that could be construed as a potential conflict of interest.

## Publisher’s Note

All claims expressed in this article are solely those of the authors and do not necessarily represent those of their affiliated organizations, or those of the publisher, the editors and the reviewers. Any product that may be evaluated in this article, or claim that may be made by its manufacturer, is not guaranteed or endorsed by the publisher.
